# Immunome profiling in prostate cancer: a guide for clinicians

**DOI:** 10.3389/fimmu.2024.1398109

**Published:** 2024-11-20

**Authors:** Luis San-Jose Manso, Arantzazu Alfranca, Ignacio Moreno-Pérez, María Ruiz-Vico, Clara Velasco, Patricia Toquero, María Pacheco, Almudena Zapatero, Diego Aldave, Guillermo Celada, Eduardo Albers, María-Dolores Fenor de la Maza, Jorge García, Elena Castro, David Olmos, Ramón Colomer, Nuria Romero-Laorden

**Affiliations:** ^1^ Urology Department, Hospital Universitario La Princesa, Madrid, Spain; ^2^ Immunology Department, Hospital Universitario La Princesa, Madrid, Spain; ^3^ Personalized Precision Medicine Chair, Universidad Autónoma de Madrid, Madrid, Spain; ^4^ Medical Oncology Department, Hospital Universitario Clínico San Carlos, Madrid, Spain; ^5^ Medical Oncology Department, Hospital Universitario 12 de Octubre, Madrid, Spain; ^6^ Medical Oncology Department, Hospital Universitario La Princesa, Madrid, Spain; ^7^ GU Translational Research Unit, Instituto de Investigación Sanitaria de la Princesa, Madrid, Spain; ^8^ Radiation Oncology Department, Hospital Universitario La Princesa, Madrid, Spain; ^9^ Medical Oncology Department, Clínica Universitaria de Navarra, Madrid, Spain; ^10^ Biocomputing Unit, Hospital Niño Jesús, Instituto de Investigación Sanitaria de la Princesa, Madrid, Spain

**Keywords:** immunome, prostate cancer, biomarkers, immunophenotype, immunotherapy

## Abstract

Tumor immune microenvironment (TIME) plays a key role to understand how tumors respond to prostate cancer (PC) therapies and potential mechanisms of resistance. Previous research has suggested that specific genomic aberrations, such as microsatellite instability (MSI) or CDK12 bi-allelic loss can allow PC patients more likely to respond to immune checkpoint inhibitors (ICI) or other immune therapies. However, responses to these treatments remain highly variable even in selected patients. Thus, it is essential to obtain more information about tumor immune cells that infiltrate these tumors, and on their plasticity and interactions, in order to better understand the underlying biology to allow development of new therapeutic strategies. This review analyzes: 1) How interactions among immune cell populations and other cells infiltrating the tumor stroma can modulate the progression of PC, 2) How the standard therapies to treat PC (such as androgen deprivation therapy, new androgen-directed hormone therapy or chemotherapy) may influence the dynamic changes of the immunome and 3) What are the limitations in characterizing the immune landscape of the host´s response to tumors.

## 1 Introduction

Prostate cancer (PC) is a heterogeneous malignancy and it is considered an immunologically “cold” tumor, unable of generating effective T-cell responses (1). It has been characterized by decreased number of cytotoxic cells and high density of regulatory T-cells (Tregs) that facilitate immune evasion. The number and proportions of immune cells with tumor immunity function, such as CD8+ T cells, NK or monocytes have been found significantly smaller in lymphatic metastases than in primary lesions of PC, implying immunosuppression in the tumor microenvironment (TME) of PC (2).

**Figure 1:**
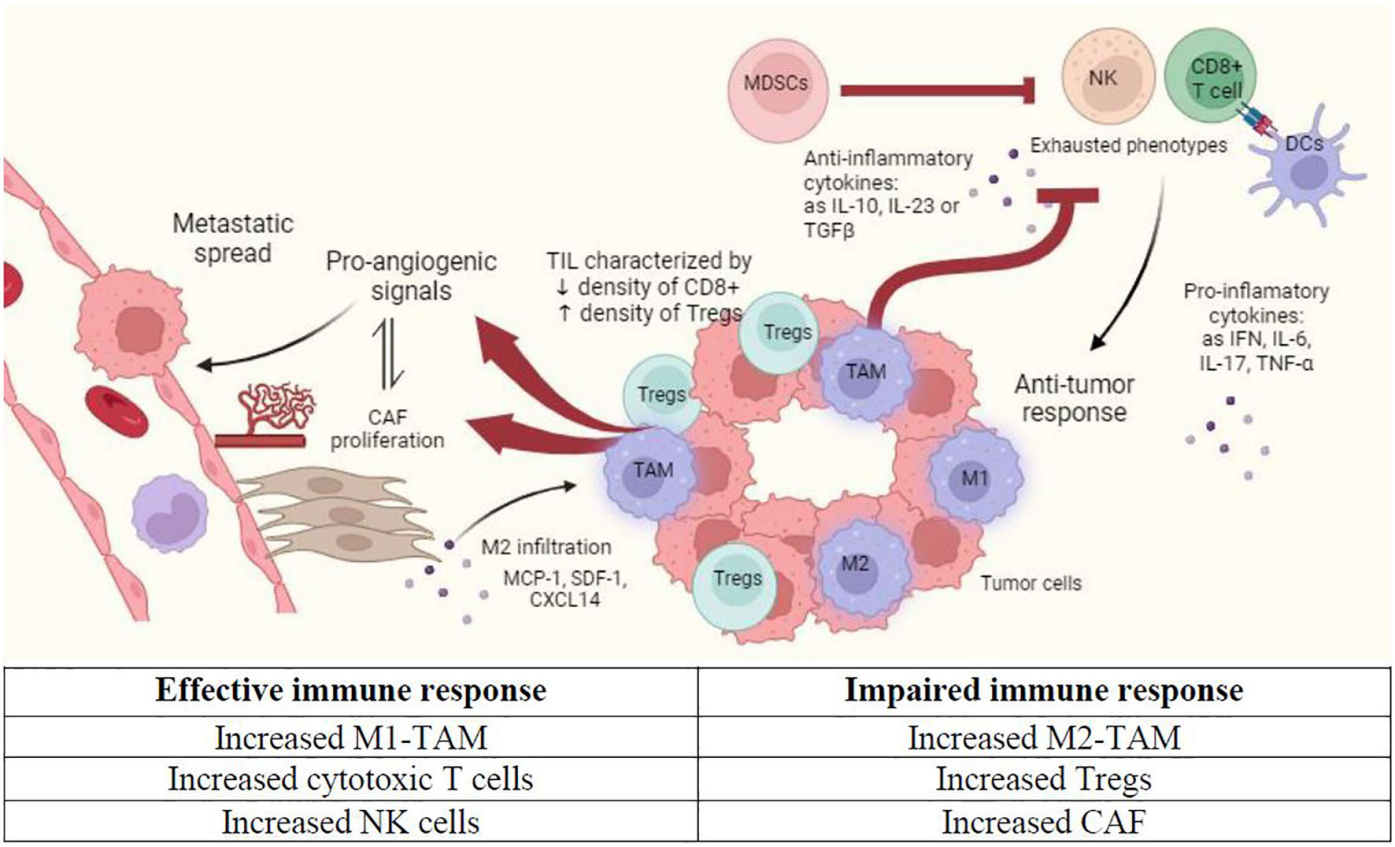
Immune microenvironment in prostate cancer (PC). This figure shows the main features that characterize tumor immune microenvironment in primary PC niche. CAF, Cancer-associated fibroblasts; DC, Dendritic cell; M1, Classically activated macrophages; M2, Alternatively activated macrophages; MDSC, Myeloid-derived suppressor cells; NK, Natural killer cell; TIL, Tumor infiltrating lymphocytes. Created with Biorender.com.

This immunosuppressive effect of PC cells may explain in part the poor results obtained by immunotherapy in advanced PC (3, 4). Peripheral blood myeloid expansion, indicated by a high neutrophil-to-lymphocyte ratio, has been associated with shorter survival (5). However, the identification of specific populations involved in immune response against the tumor, and their respective role and cell-cell interactions remain unclear. Below, we describe the immune microenvironment that characterizes PC (**Figure 1**, **Table 1**).

**Table 1:** Immune cell subpopulations and their role in PC.

Cell Populations	Subtypes		Immune Response and PC	Regulatory cytokines/proposed therapeutic target
Monocytes and TAM	M1 TAM (anti-tumorigenic		• Promotes pro-inflammatory environment	IFN-γ, TNFα
	M2 TAM (pro-tumorigenic)		• Favoring metastatic spread • Promote *de novo* angiogenesis	IL-6, IL-10, TGFβ
	Monocyte-macrophage (transitional cell state)		• Higher peripheral blood monocyte counts associated with aggressive PC	CSF1, CXCR2
Cancer-associated fibroblasts			• Induce inflammation • Stimulate M2 macrophage infiltration	MCP-1, CCR2
Tumor-infiltrating lymphocytes	CD4+ T cells	Th1 Th1 Th17 Tregs T naive	• Tregs associated with increased risk of cancer recurrence	CCR4 Co-inhibitory receptors in exhausted phenotypes (PD-1, LAG3, TIGIT)
	CD8+ T cells	CTL-1 CTL-2 CD-8 effector	• Reduced CD8+: poor prognosis and shorter time to biochemical recurrence after radical prostatectomy	
Natural killer	NKT CD56dim CD56bright IL7R+ CD56bright IL7R-		• Elevated NK cell expression in tumors associated with lower risk of recurrence • In peripheral blood, detection of NK cells have been correlated with PC diagnosis	CXCR4, MMP-9 TGFβ1 and PGE2 involved in exhausted phenotypes
Myeloid-derived suppressor cells	PMN-MDSC: CD14+ M-MDSC: CD15+		• Promotes immune suppression • Higher levels of circulating MDSC associated to worse overall survival	IL-23, CXCR2, CCL20-CCR6 axis
Dendritic cells	Classical DC		• Stimulate T-cell responses and NK cells	Vaccines using autologous DC
	Plasmocytoid DC		• Plasmocytoid DC produce IFN that provide effective co-stimulation for naïve and memory B cells	

TAM, Tumor-associated macrophages; MDSC, Myeloid-derived suppressor cells; DC, Dendritic cells; CSF1 or M-CSF, Macrophage colony-stimulating factor; MCP-1, Monocyte chemotactic protein-1; SDF-1, Stromal-derived growth factor-1.

## 2 Immune phenotype in response to prostate cancer

An immune suppressive tumor microenvironment associated with suppressed myeloid populations and exhausted T-cell has been described (6). Despite detailed characterization of the epithelial and tumor cells, the immune phenotype in different clinical stages of PC is not well known.

### 2.1 Monocytes and tumor-associated macrophages

CD68+ tumor-associated macrophages (TAM) are the most abundant cell type in the TME of PC (7). Macrophages are a plastic immune population that can take on a wide range of phenotypes, ranging from anti-tumorigenic (M1, classically activated) to pro-tumorigenic (M2, alternatively activated). This fact makes identifying them correctly challenging. Recent studies based on RNA sequencing have revealed a third subpopulation annotated as monocyte-macrophage that suggests a transitional state from monocytes toward macrophages (6).

Classical M1 TAM carry out pathogen clearance by secreting pro-inflammatory factors such as IL-1β, IL-12, IFN-γ and TNF-α. They are characterized by higher levels of reactive oxygen species (ROS), inducible nitric oxide synthase 2 (iNOS), and major histocompatibility complex (MHC) class II. Conversely, M2 TAM are increased in the context of hypoxia, and high levels of IL-6 or IL-4, and these cells express autocrine factors such as TGF-β and IL-10 to promote their own maturation. Non-coding RNA and several transcription factors including interferon (IFN) regulatory factors and c-Myc, have been shown in M2 activation (8).

Macrophages depend on stimulation by different cytokines, hormones such as glucocorticoids, signaling extracellular vesicles, and extracellular matrix components (9). A prominent cytokine known to regulate macrophage differentiation and proliferation is the macrophage colony-stimulating factor (M-CSF or CSF1), which has been proposed as a therapeutic target (10). Other chemokines, like the granulocyte colony-stimulating factor (G-CSF) and chemokine ligand 2 (CCL-2), whether secreted from tumor or stromal host cells, are associated with TAM recruitment. Recently, macrophage receptor CXCR2 has been proposed as a major driver of TAM polarization, and a new therapeutic target in PC (7, 11, 12). In a feedback loop, macrophages also contribute to overproduction of chemokines (9).

M2 macrophages are thought to be related to cancer-associated fibroblasts (CAF). CAF are active recruiters of monocytes to tumor cells. They act through stromal-derived growth factor-1 secretion and promote trans-differentiation to M2. TAM and CAF cooperate in increasing tumor cell motility, to favor the escape of cancer cells from the primary site and their metastatic spread. In addition, TAM and CAF promote activation of endothelial cells and their bone marrow-derived precursors in *de novo* angiogenesis (13). CAF induce inflammation and stimulate macrophage infiltration via secretion of cytokines such as monocyte chemotactic protein-1 (MCP-1), stromal-derived growth factor-1 (SDF-1), CXCL14 and CCR-2. Clinical studies have shown the importance of CAF in biochemical recurrence and metastasis for patients undergoing radiotherapy or radical prostatectomy (14).

In peripheral blood, elevated monocyte counts have been correlated with aggressive tumor features and poor survival in patients with castration resistant prostate cancer (CRPC). However, studies in early stages have not found any association between monocyte counts and biochemical recurrence, CRPC or PC-specific mortality (15–17).

### 2.2 Tumor-infiltrating lymphocytes

Tumor-infiltrating lymphocytes (TIL) are key mediators of anti-tumor immune response given their ability to recognize peptides/MHC class I complexes through T-cell receptors (TCRs). The presence of cytotoxic and T helper cells within tumor margins has been associated with favorable prognosis across multiple cancer types (18).

However, in PC, an increase in T-cell infiltration has been frequently correlated with poor prognosis and shorter time to biochemical recurrence after radical prostatectomy (19, 20). This may be attributed to PC showing reduced lymphocyte infiltration, especially of the CD8+ cytotoxic subset, due to the hypoxic environment. In addition, CD8+ cells may have impaired cytotoxic responses despite tumor antigen stimulation (20). These so-called “exhausted” T-cells (Tex) are characterized by increased expression of coinhibitory receptors, the best known of which is PD-1, but also LAG3, TIM3, and TIGIT (21, 22).

Preclinical studies have shown that Tregs can suppress anti-tumor responses, which are directly related to an increased risk of cancer recurrence (23). Preliminary data suggest that the presence of other receptors such as CCR4 on Tregs may impact PC patient survival, although further research is required (24).

Recent studies based on RNA sequencing have described up to four CD4+ T-cell subpopulations (Th1, Th17, Tregs and Tnaïve) and three CD8+ T-cell subpopulations(CTL-1, CTL-2 and CD-8 effector) based on functional scores (6). These differences in the classification and the functional role of each TIL subtype are important to consider in future studies on immunome in PC.

### 2.3 Natural killer cells

Prostate tissue is generally poorly infiltrated by natural killer (NK) cells. Elevated NK cell expression in prostate tumors is associated with a lower risk of disease recurrence, as measured by biochemical recurrence after radical prostatectomy (25, 26).

NK cells infiltrating prostate tissues display unexpected immature features with decreased expression of activating receptors such as NKG2D and membrane proteins such as CD16, which are involved in antibody-dependent cellular cytotoxicity. The presence of this phenotype may be mediated by the action of TGFβ1 and PGE2, that would lead to the expression of immature NK cells with low cytotoxic potential and a hyposensitive functional status against target cells (27).

Four NK subpopulations have been described based on gene expression: NKT, CD56dim, CD56bright IL7R+ and CD56bright IL7R-. NKT cells are characterized by high expression of T-cell marker genes. CD56dim NK cells have high expression of HAVCR2. CD56bright NK cells are segregated in two subtypes depending on IL7R expression and the homing-receptor SELL (CD62L). Of the NK subpopulations, CD56dim cells score highest for exhaustion and are in higher abundance in PC compared to benign prostate samples (6).

In peripheral blood, detection of NK cells may play a role in early diagnosis. Barkin et al. observed in a small cohort (n=43) that patients with low levels of NK cells were more likely to have a positive prostate biopsy result (28). Recently, PC peripheral NK cells have been linked to enhanced CD9, CD49a, CXCR4, CXCL8, MMP-9 production, monocyte-recruiting and polarizing factors (29).

### 2.4 Myeloid-derived suppressor cells

Myeloid-derived suppressor cells (MDSC) are pathologically activated neutrophils and monocytes with potent immunosuppressive activity. They are classified as polymorphonuclear (PMN-MDSC: CD14+) or monocytic (M-MDSC: CD15+) and they can allow immune system evasion by inhibiting T-cell, B-cell and NK-cell mediated immune responses (30).

Higher levels of circulating MDSC have been linked to a worse overall survival in PC, and therefore the detection of this cell type has attracted a lot of attention (31). Previous studies have shown, using PC mouse models, that intratumor myeloid cells can drive paracrine oncogenic signaling, senescence evasion, and immunosuppression (7, 11, 32). High levels of PMN-MDSC in metastasis have been associated with higher levels of MDSC-recruiting chemokines in those areas, such as CXCL5/CXCR2 signaling (33). CXCR2 has been proposed as a new therapeutic target. A proof-of-concept trial with CXCR2 inhibitors has shown positive results with durable clinical benefits in terms of biochemical and radiological responses in patients with metastatic CRPC (12).

The inflammatory cytokine IL-23 produced by MDSC has been recently linked to CRPC development, since it induces the transcription of AR target genes through STAT3 transcription factor, leading to the proliferation of cancer cells and tumor survival (34). Thus, the IL-23 and STAT3 pathways have been proposed as new therapeutic targets in this setting (35, 36).

In bone metastasis, the chemokine CCL20 is overexpressed by myeloid cells, as is its CCR6 receptor on T cells. Disruption of CCL20-CCR6 axis in preclinical models restores T cell reactivity and improves survival, making it another attractive drug target linked to MDSC (37).

### 2.5 Dendritic cells

Dendritic cells (DC) are a heterogeneous group of antigen-presenting cells that can be classified into two basic subtypes: plasmacytoid DC, which accumulate in blood and lymphoid tissue, and classical DC, which infiltrate lymphoid and nonlymphoid tissues. However, plasmocytoid and classical DC markers can change in certain situations, such as inflammation or infection, making their identification more difficult (38).

DCs have a high capacity to induce and stimulate T-cell responses and improve the cytotoxic potential of NK cells, thus contributing to the elimination of tumor cells. For that reason, they have been considered a notable therapeutic target for the development of vaccines using autologous DC-based immunotherapy (39, 40). Sipuleucel-T, a DC based immunotherapy, improved overall survival in PC (39). Despite this initial success, there was controversy around a particularly unfavorable outcome of the control arm. Furthermore, subsequent DC vaccines have failed to produce significant benefit, and the utility of this strategy remains unproven.

### 2.6 Soluble immune-related factors in the tumor microenvironment

Multitude of soluble immune-related factors are secreted by immune cells within TME, as well as by the tumor itself, contributing to a complex landscape. Krueger et al. proposed that mesenchymal stem cells found in radical prostatectomy specimens may promote tumor progression by regulating the immunosuppressive microenvironment, suppressing T-cell proliferation and secreting soluble factors (TGFβ, IL-6, IL-10, IDO, among others) (41).

Nakashima et al. found that serum IL-6 correlated significantly with the clinical stage of PC (42). High expression of TGF-β and IL-6 in tissue has been also associated with poor prognosis (43). IL-10 has anti-inflammatory functions and supports tumor progression by limiting efficient antitumor response. It has been found to be related to Gleason score and considered a poor prognostic factor (44).

Different levels of TNF-α and IFN-γ have been associated with PC progression. High levels of TNF-α lead to tumor cell necrosis and apoptosis, contributing to immune cytotoxicity and release of other inflammatory cytokines. Paradoxically, a low dose paracrine TNF-α production in tumor areas may contribute to chronic inflammation and cancer progression. PC cells have been shown to be poorly sensitive to effects of IFN-γ. Furthermore, it has been suggested that IFN-γ could induce immunosuppressive effects in PC (45). Indoleamine 2,3-dioxygenase (IDO) is a tryptophan-depleting enzyme that has been extensively investigated due to its role in inhibiting the expansion of T-cells, suppressing adaptive immune responses. Evidence has shown that IDO and the tryptophan pathway are linked to immune tolerance (46). More recently, IDO has been shown to induce resistance to treatment with ICI, upon stimulation by pro-inflammatory cytokines, mainly IFN-γ (45).

## 3 Immunome response after prostate cancer therapy

The following section describes how the immune landscape evolves after the most common treatments for PC (**Table 2**).

**Table 2:** Summary of evidence reporting changes in TIME with standard therapies used in advanced prostate cancer.

Treatment	TIME changes related to therapy		References
	In the tumor	In peripheral blood	
ADT	• Increased T-cell infiltration • Decreased pro-inflammatory cytokines such as IL-1, IL-6 and TNFα levels • Increased TAM	• Increase in CD4 naïve subpopulation • Decrease in NK cell activation markers	(25, 47–50)
ARSI	• Increased pro-inflammatory mediators, including IFN-γ, IL-5 and TNF-α. • Higher expression of PD-L1/2 positive dendritic cells • Promotion of MDSC	• Increased levels of pro-inflammatory cytokines such as IFNγ, IL-5, IL-10, TNFα • Increase in antigen specific T-cells levels targeting PSA • Decrease in MDSC	(51–54)
Docetaxel	• Increased T-cell infiltration • Induction of IFN signaling • Increased T memory lymphocytes • Decreased T regs • Upregulated PD-L1		(55)
Radiotherapy	• Stimulates pro-inflammatory cytokines. • Increase in T-cell infiltration • Upregulation of MHC-I • Increased CTLA4, PD-L1/L2, TGFβ • Activation of CSF1 signaling stimulation of macrophage recruitment.		(56–58)

ADT, Androgen deprivation therapy; ARSI, Androgen receptor signalling therapy; TAM, Tumor-associated macrophages; MDSC, Myeloid-derived suppressor cells.

### 3.1 Androgen deprivation therapy

Androgen inhibition has been shown to result in thymic volume regeneration and enhanced T-cell lymphocyte production (59). *In vivo* studies have also linked ADT to a proliferative response to antigen-specific T-cell stimulation, faster lymphocyte recovery following chemotherapy, and T-cell infiltration into the prostate (60, 61). Under ADT, a prospective cohort of 20 patients showed an increase in the naïve CD4 subpopulation (CD45RO-/CCR7+) in peripheral blood after therapy. The other subpopulations (CD4+ and CD8+ effector, central memory, or effector memory) remained relatively stable over time. It suggests that ADT not only affects the frequency of T-cell subsets, but also their responsiveness to proliferation through co-stimulatory molecules (47). Other studies have also described how ADT induces CD4+ and CD8+ T-cell infiltration in PC compared to normal prostate tissue, which could be detected within the first month of treatment (48).

Although this may suggest a better response to immunotherapy in patients treated with ADT, it has been refuted by later studies. Furthermore, a decrease in inflammatory cytokines such as IL-1 IL-6, IL-8 and TNFα levels after initiation of ADT has been identified, with no effect on IFN levels (49, 50). These changes could potentially facilitate tumor growth by immune system suppression (62). An increase of CD68+ macrophages within the tumor has also been reported (26).

NK cell markers have been linked to prognosis in patients undergoing ADT (25). NK cells from metastatic PC patients with longer response to castration display phenotypic and functional patterns associated with high expression of activating receptors and molecules involved in NK cell maturation and degranulation.

In terms of progression to castration resistance, a key role for macrophages is emerging. El-Kenawi et al. demonstrated in a mouse PC model how macrophages took up cholesterol in the form of low-density lipoprotein and transported it to the cancer cells to support the synthesis of androgens. Macrophages also seemed to be able to stimulate AR translocation to the nucleus. Furthermore, they demonstrated that macrophage density correlated with ADT resistance and that macrophage depletion in mice (using a CSF1 antibody) reduced both tumor androgen levels and various surrogates of AR activation, as well as better outcome after ADT (63). This mechanism has been the basis for statin trials in PC patients under ADT.

### 3.2 Androgen receptor signalling inhibitors

Metastatic CRPC patients treated with abiraterone or enzalutamide have shown significantly lower levels of fibroblast growth factor, granulocyte-macrophage colony-stimulating factor (GM-CSF), IL-10 and IL-6 in plasma in ADT + ARSI sensitive compared to *de novo* resistant patients (51). Increased levels of IFNγ, IL-5, IL-10, TNFα and the chemokine macrophage inflammatory protein 1 alpha (MIP-1a)/CCL3 at week 8 after ARSI initiation suggest potential activation of T-cell-mediated immune responses to abiraterone/enzalutamide. The authors hypothesized that these immune markers could predict outcomes in patients treated with ARSIs.

Furthermore, *in vivo* studies showed how enzalutamide promotes MDSC-mediated immune suppression and tumor growth, leading to metabolism changes through the MPC-2 and MAPK pathways. This may be based on decreased mitochondrial respiration and increased glycolytic rate, showing the dynamic interactions between tumor, stroma, and immune cells. Therefore, it suggests a mechanism of ARSI resistance based on enhancement of myeloid tumor-promoting activity (52).

More recently, Madan et al. presented their results regarding the immunologic impact of enzalutamide in non-metastatic hormone sensitive PC patients. The study involved 38 patients treated with short-course enzalutamide, and found a rise in antigen specific T-cell levels targeting PSA and NK cells, plus a decrease in MDSC in blood (53). However, no significant association was found between clinical responses and immune changes, perhaps due to the small number of patients studied.

In metastatic CRPC treated by enzalutamide, a higher expression of PD-L1/2+ DC was identified compared with treatment-naïve or responder patients. This was suggested as a non-AR driven hyphen mechanism of resistance by the authors (54). In addition, PD-L1+ circulating tumor cells are more frequent in metastatic CRPC patients progressing to ARSI than those starting ARSI. This study also addressed heterogeneity in the frequency of immune related biomarkers such as PD-L1/L2, CTLA-4, and B7-H3, which could potentially account for the poor outcomes with ICI in metastatic unselected PC patients (64).

Macrophages have also been shown to be involved in resistance to ARSI. In metastatic CRPC patients, abiraterone- or enzalutamide-sensitive patients exhibited increased pro-inflammatory mediators, including IFN-γ, IL-5, and TNF-α, which were generally identified as M1 markers (56). Studies in PC models have shown how macrophages directly regulated AR nuclear translocation and resistance to enzalutamide (63). Inhibitors of CSF1 signaling have been tested in combination with abiraterone demonstrating that TAM blockade in this setting disrupted tumor promotion and maintained a more durable therapeutic response compared to abiraterone alone (10).

In peripheral whole blood, RNA deconvolution analysis from metastatic CRPC patients treated with enzalutamide (n=226) revealed that progression to this therapy was correlated with expansion of monocytes and contraction of CD8 lymphocytes (65).

### 3.3 Chemotherapy

Docetaxel regulates several immune-related pathways. T-cell (IFNγ and TNFα gene sets), B-cell, and NK cell mediated immunity are strengthened after docetaxel therapy, with an increase in CD8+, CD3+, and CD4+ T-cells, and a decrease in regulatory T-cells, mainly through the cGAS/STING-IFN pathway. Docetaxel-based chemohormonal therapy upregulated PD-L1 (55).

Baseline neutrophil-to-lymphocyte ratio and the systemic immune-inflammation index has proven to be related with worse prognosis in patients with mCRPC treated with docetaxel (15, 66, 67).

Preclinical studies have shown that chemoresistance to docetaxel is mediated by CCL5 cytokine secreted by CD4+, which can increase the aggressive potential and stem cell populations of PC. Also, this could potentially activate the PI3K/Akt and STAT3 pathways, which could be related to aggressiveness and cell migration (68). Baseline IL-6 levels have been inversely correlated with response, time-to-progression, and overall survival in docetaxel-treated patients (69). However, clinical attempts to demonstrate the efficacy of IL-6 antibodies in this setting have failed (70).

Preclinical studies have found that TAM promoted the survival of PC cells after docetaxel via the CSF1/CSF1R-CXCL12/CXCR4 axis in CRPC. The combination therapy of docetaxel and CSF1 inhibitors is currently under study (71). Regarding cabazitaxel, the CCL2-CCR2 axis has been found to be a key contributor to cabazitaxel resistance in CRPC (72).

Clinical studies have shown the emergence of polyaneuploid cancer cells, able to resist stress within the TME, as a survival strategy for the PC population. These cells correlate with poor response to docetaxel chemotherapy in the context of CRPC (73).

### 3.4 Radiotherapy

Radiotherapy (RT) has been shown to initiate a pro-inflammatory cascade and to increase systemic response rates to immunotherapy. Abscopal effect, although anecdotal, has been described in patients treated with RT and ICI (74). High-dose RT stimulates pro-inflammatory cytokines, such as TNFα, IL-1, IL-6, and IL-8, increasing the presence of activated T-cells; upregulation of MHC class I molecules to facilitate antigen presentation; and the presence of effector cells through the production of chemotactic chemokines and the upregulation of vascular adhesion molecules (75). Low-dose RT, additionally, can overcome immune desert tumors by increasing T-cell infiltration in TME (57).

In a series of 48 localized PC patients, several immune cell populations (Th1-cells, B-lymphocytes, CD8+ T-cells, Tregs, and TAM) were measured in tissue samples before and after treatment (with ADT or RT monotherapy or both). Overall, an increase in at least 3 over 5 of the immune populations were documented in all three arms. RT alone increased levels of Th1, B-cells or Tregs whereas RT in combination with neaoadjuvant ADT increased all subsets (76).

Keam et al. analyzed the effects of high dose brachytherapy on immune response mediators in localized PC (n=24). They found that many immune checkpoint molecules, such as CTLA-4, PD-L1 and PD-L2, as well as TGFβ levels, were increased in response to radiation, thus turning an immunologically “cold” tumor into a “hot” tumor, which may translate into better response to immunotherapy (58).

Clinical and preclinical studies have suggested that local irradiation induces ABL1-dependent CSF1 production, followed by activation of CSF1/CSF1R signaling leading to systemic macrophage recruitment (56).

### 3.5 Response to immune therapies

Only 10–30% of unselected patients respond to PD-1/PD-L1 blockade. However, it has been reported that those patients with MSI, around 3% of total PC, correlate with high tumor mutation burden, and subsequently, responses under ICI. This data led to an agnostic approval for MSI cancer patients. Interestingly, it has been suggested that in some of those patients, the MSI phenotype could be acquired by dynamic changes in TME and response to previous therapies (77). Delving into the underlying mechanisms could be crucial to improve current results of IT in PC.

There are no other predictive biomarkers for ICI in PC. The expression of PD-1 in lymphocytes is highly prevalent in PC compared with benign tissues and it has been associated with Gleason score. However, it has not been proven to be useful as a marker to select patients who would respond to ICI and its independent role in PC survival is yet to be validated (19, 78).

Combinations with anti-CTLA-4, chemotherapy, ARSIs, radionuclides and PARP inhibitors have been explored in phase I-III trials with negative results. Presence of androgen receptor splice variant 7 and alterations in DNA repair genes showed higher response rates, but this was not translated into better survival (62). Studies with (177)Lu-PSMA-617 and ICI are ongoing, based on immune-modulation of this drug and promising results of phase I trials (79). Loss of PTEN tumor suppressor is associated with poorly T cell-infiltrated tumors and resistance to anti-PD-1 immunotherapy, being present in 40-50% of primary and 70-90% of metastatic PC. Preclinical studies with intermittent PI3K inhibitors have shown promising results to overcome resistance to ICI (80).

Accumulated studies confirm that hypoxia potently induces HIF-1α-dependent PD-L1 expression on tumor cells, suggesting that PD-L1 expression can be upregulated in hypoxic tumor cells to promote immune escape from cytotoxic T cells. It has been suggested that co-blockade of PD-L1 and HIF-1α signaling might represent a promising approach to enhance the activity of cytotoxic T cells (81). However, combination of antiangiogenesis and ICI has been tested in phase III trials with differences in progression-free survival but no impact in OS (82).

Chimeric antigen receptor T cells (CAR-T) therapies targeting PSMA to re-direct the action of T cells have shown promising efficacy in preclinical studies, with Phase I-II trials ongoing (83). Bi-Specific T cell enhancers (BITE) bind simultaneously to immune cell markers (such as CD3 for T-cells or CD16 for NKs) and tumor-associated antigens to re-direct and potentiate the immune response. BITE immunotherapies targeting PSMA are currently being studied in early-stage clinical trials. Combination of ICI and BITE has shown preliminary efficacy in metastatic CRPC patients (84). Identifying the optimal moment to act on the immunome at each stage of the disease and in response to previous treatments may be crucial for the success of these new therapies.

## 4 Limitations to study TIME in prostate cancer

### 4.1 Main barriers

PC heterogeneity remains a challenge up to date, and currently we know that in addition to genetic factors, this diversity is derived also from the environmental landscape including fibroblast recruitment, immune cells migration, matrix remodeling, angiogenesis role and epigenetic influence (81). Moreover, TIME is dynamic with spatial and temporal changes in response to anticancer therapies. In fact, the continuing crosstalk between tumor cells and TME is fundamental for cancer progression, including therapeutic resistance. To capture this variability is one of the more important barriers when studying TIME in solid tumors. It may be improved if technical approaches to study immune response in peripheral blood are implemented, but validation and correlation with TME scenario in the primary tumor and metastasis is needed first. Moreover, to identify rare immune subsets is still challenging, and may lead to bias in data interpretation.

Recent studies have also provided a new perspective to understand antitumor immunity, which can be initiated directly at the tumor site or metastasis in spatially well-organized areas of infiltrative immune cell aggregates called tertiary lymphoid structures. These ectopic lymphoid structures have been correlated with prognosis in different tumors, with the potential of being modulated by different therapeutic strategies (85).

Beyond tumor heterogeneity, we have to consider the heterogeneity between individuals, since the immune response will depend on the characteristics of our body, such as age or the pre-existence of systemic inflammatory processes. Thus, studies published with matched-control normal biopsies that include a small number of controls may not be representative of the heterogeneity among healthy individuals (6, 86). Moreover, there is a lack of data about the immune environment in normal prostate tissue or pre-neoplastic lesions. All these factors should be included for PC TIME study design.

Another notable barrier to describe TIME is clonal heterogeneity, as PC is a multifocal disease and each tumor focus might have different phenotype. The impact of TIME on cancer cells is characterized by the regional heterogeneity in hypoxia, acidity and cytokines within the tumor environment. Intriguingly, many type of cells (such as CAF or TAM) possess tumor suppressive effects or tumor promoting effects depending on the dynamic interaction into the TME.

Different attempts to classify patients into different subtypes has been performed, but their utility in clinical practice remains controversial because they do not correlate precisely with response to standard therapy (87, 88). Recently, Weiner et al. published a molecular classification using gene expression, TIL data based on immunohistochemistry (IHC) and clinical characteristics, and they found 4 clusters: luminal differentiated, luminal proliferating, basal immune, and basal neuroendocrine. Tumors with the basal immune subtype represented 35.1% of the total sample and they were characterized by a significant IFN activity and increased TIL. However, no specific targeted therapy has been described for this subgroup (89).

### 4.2 Technical approaches

Beyond the simplicity of the Immunoscore test, additional data about the spatial location of the infiltrate and the interactions between different cell types and stroma are needed to understand the complexity of PC TIME. New approaches are being developed such as optimized multiplexing panels for immunofluorescence and the use of machine learning models. These advances allow for a precise definition of relevant patterns in terms of spatial clustering of the intratumoral immune infiltrate, and the association with clinical parameters such as survival or response to treatment. However, the algorithms developed must be carefully validated in independent cohorts.

Regarding expression analysis, first studies using arrays showed that immune response plays a key role in advanced PC (90). Soon after that, next-generation sequencing (NGS) obtained a complete immune landscape from high-throughput genomic data. NGS allows for bulk populations to be analyzed using deconvolution algorithms in order to infer the cell types present in the sample (such as xCell or EPIC). These signatures-based method have been developed from knowledge of thousands of pure cell types from various sources (14, 91). NGS, however, relies on the prior existence of known gene expression profiles, and does not provide information on the degree of heterogeneity of the population.

Classically, strategies have been used to analyze data obtained with different techniques separately and integrate them at a later stage, which usually implies that the data come from different cells within the sample. But the ideal integration system should aim at simultaneous acquisition of data derived from the same cell, to try to explain the fluidity and heterogeneity of the TIME in PC. For that reason, strategies based on integrated single-cell(sc) analysis are promising in this area (92).

RNA levels, however, do not always correspond directly with the presence of the protein, and can therefore provide misleading information about gene expression levels. Techniques such as CITE-Seq have been developed that allow for simultaneous detection of mRNA and protein levels in each cell, combining specific oligonucleotide-labeled antibodies against surface proteins with transcriptomic analysis.

At a transcriptional level, Han et al. have published the Tumor Immune Contexture Score (TICS), describing expression signatures related to immune microenvironment in patients with localized PC, that have shown a prognostic value of this RNA-based biomarker independent of TNM stage, PSA and Gleason score (93). A high TICS showed prolonged biochemical recurrence-free survival after radical prostatectomy. Previously, other authors had published RNA signatures in whole blood from patients with CRPC involving genes that suggested a possible dysregulation of the immune system. Immunogenic signatures have also been linked to identify the most lethal subtypes in PC (90, 94, 95).

Recently, an integrative analysis of TIME in metastatic CRPC has been published (n=100). Metastatic biopsies were analyzed using whole exome RNA sequencing, measuring tumor mutational burden and T-cell–inflamed gene expression profile score (Nanostring). IHC for PD-L1, ATM, PTEN, SOX2, and the presence of neuroendocrine features were also studied. The authors found that PD-L1, TcellinfGEP score, and SOX2 had prognostic value in this setting (96).

## 5 Discussion

**Figure 2:**
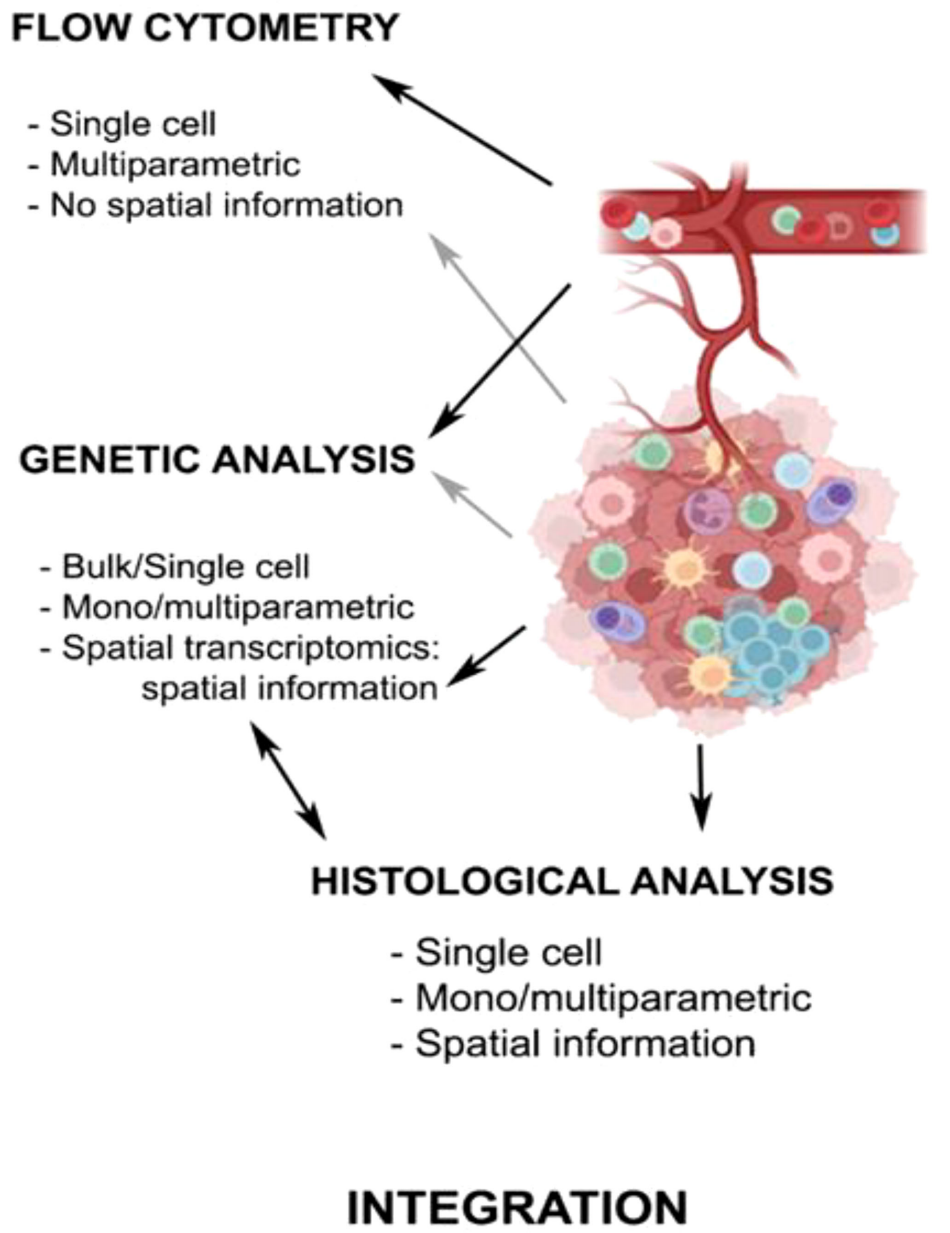
Technical approaches to analyze antitumor immune response. The chart summarizes the main characteristics of the techniques used in the study of the TIME in PC, with either peripheral blood or tumor samples. Created with Biorender.com.

The integration of the information obtained from different approaches (transcriptomic, genomic, epigenomic, proteomic, and topographic data) is of great relevance to obtain a complete picture of the landscape of the anti-tumor immunome (**Figure 2**) (97). Functional studies based on gene expression are currently indispensable to classify correctly different immune subpopulations and understand the dynamic changes in the TIME.

Analysis of tumor samples from the selected small subgroup of PC patients who responded to anti PD-1/PD-L1 antibodies has revealed a specific phenotype with the presence of cytotoxic T-cell infiltrates, high PD-L1 receptor expression, and a higher mutational burden or MSI (98). However, the vast majority of PCs do not display these features. They show an immunosuppressive microenvironment mainly comprised of MDSC, TAM, and different soluble factors (TGF, IL-6/10/23) as main mediators of the downregulation of the innate immune response (41). This status frequently leads to immunological tolerance, a state of unresponsiveness of the immune system to the tumor. This could partly explain the lack of efficacy seen with ICI in molecularly unselected PC. Therefore, new strategies to overcome this dialed down turned-off immune system must be developed, with synthetic strategies based on BITE tested in clinical trials (99, 100).

In conclusion, the potential to discover new therapeutic approaches to improve PC outcomes relies in part on a better understanding of each step of the immune cycle, providing more accurate mechanistic insight (21). Many questions remain about how the immune system modulates, and how it can be modulated by the current standard therapies, thereby impacting PC progression. Therefore, further research integrating genomic, molecular, cellular, histologic and functional data is necessary to overcome these limitations.
